# **Functional enhancement of mesothelin-targeted TRuC-T cells by a PD1**-**CD28 chimeric switch receptor**

**DOI:** 10.1007/s00262-023-03556-7

**Published:** 2023-10-18

**Authors:** Derrick McCarthy, Michael Lofgren, Amy Watt, Holly Horton, Philippe Kieffer-Kwon, Jian Ding, Sebastian Kobold, Patrick A. Baeuerle, Robert Hofmeister, Dario A. Gutierrez, Robert Tighe

**Affiliations:** 1 TCR2 Therapeutics, Inc., 100 Binney Street, Suite 710, Cambridge, MA 02142 USA; 2 Adaptimmune, Philadelphia, PA USA; 3 Center of Integrated Protein Science Munich (CIPS‑M) and Division of Clinical Pharmacology, Department of Medicine IV, Member of the German Center for Lung Research (DZL), University Hospital, Ludwig-Maximilians-Universität, Munich, Germany; 4 German Cancer Consortium (DKTK), Partner Site Munich, Munich, Germany; 5 Institute of Immunology, Ludwig-Maximilians-Universität, Munich, Germany

**Keywords:** T cell receptor fusion construct T cells, Solid tumor, Mesothelin, PD-1, PD-L1, CD28

## Abstract

T cells expressing a mesothelin (MSLN)-specific T cell receptor fusion construct (TRuC^®^), called TC-210, have demonstrated robust antitumor activity in preclinical models of mesothelioma, ovarian cancer, and lung cancer. However, they are susceptible to suppression by the programmed cell death protein 1 (PD-1)/programmed cell death protein ligand 1 (PD-L1) axis and lack intrinsic costimulatory signaling elements. To enhance the function of anti-MSLN TRuC-T cells, chimeric switch receptors (CSRs) have been designed to co-opt the immunosuppressive PD-1/PD-L1 axis and to deliver a CD28-mediated costimulatory signal. Here, we report that coexpression of the PD1-CD28 CSR in TRuC-T cells enhanced T cell receptor signaling, increased proinflammatory effector cytokines, decreased anti-inflammatory cytokines, and sustained effector function in the presence of PD-L1 when compared with TC-210. Anti-MSLN TRuC-T cells engineered to coexpress PD1-CD28 CSRs comprising the ectodomain of PD-1 and the intracellular domain of CD28 linked by the transmembrane domain of PD-1 were selected for integration into an anti-MSLN TRuC-T cell therapy product called TC-510. In vitro, TC-510 showed significant improvements in persistence and resistance to exhaustion upon chronic stimulation by tumor cells expressing MSLN and PD-L1 when compared with TC-210. In vivo, TC-510 showed a superior ability to provide durable protection following tumor rechallenge, versus TC-210. These data demonstrate that integration of a PD1-CD28 CSR into TRuC-T cells improves effector function, resistance to exhaustion, and prolongs persistence. Based on these findings, TC-510 is currently being evaluated in patients with MSLN-expressing solid tumors.

**Supplementary Information:**

The online version contains supplementary material available at 10.1007/s00262-023-03556-7.

## Introduction

We have engineered a novel adoptive T cell therapy platform by integrating a T cell receptor fusion construct (TRuC^®^) into the T cell receptor (TCR). This leverages the full signaling capacity of the TCR in a human leukocyte antigen (HLA)-independent manner and addresses the limitations of chimeric antigen receptor engineered T (CAR-T) cells and TCR-engineered T (TCR-T) approaches. TRuC-T cells consist of a tumor antigen binding domain fused to the CD3ε subunit of the TCR complex, which upon its integration into the TCR redirects T cell killing against tumor cells. This novel design has shown functional advantages over CAR-T cells in preclinical models, including faster tumor regression, lower cytokine production, increased solid tumor infiltration, increased oxidative metabolism, and enhanced persistence [1]. Based on promising preclinical evidence, a Phase 1/2 clinical trial examining TC-210 (*gavocabtagene autoleucel [gavo-cel]*) is ongoing in patients with advanced mesothelin (MSLN)-expressing cancer (NCT03907852).

The tumor microenvironment (TME) of solid tumors presents a major hurdle in realizing the full potential of T cell therapies, as immunoinhibitory molecules are often abundant, and positive costimulatory molecules are lacking [2]. Overexpression of programmed cell death ligand (PD-L)1 and PD-L2 on tumor cells directly inhibits T cell function by activating the programmed cell death protein 1 (PD-1) [3].

Furthermore, full T cell activation requires TCR recognition of cognate peptide major histocompatibility complexes (MHCs) (signal 1) in conjunction with costimulation, driven most prominently by activation of CD28 (signal 2) [4]. A lack of sufficient costimulatory signaling leads TCR-activated T cells to enter a hyporesponsive state known as anergy [5].

Suppression by the PD-1/PD-L1 axis-mediated suppression within the TME and the lack of intrinsic CD28 signaling afforded by the TRuC construct may present hurdles to optimal TRuC-T cell efficacy. Thus, we engineered and preclinically tested chimeric switch receptors (CSRs) designed to co-opt the immunosuppressive PD-1/PD-L1 axis and, at the same time, deliver a CD28-mediated costimulatory signal. CSRs are designed to convert a normally immunoinhibitory interaction into an immunostimulatory event by genetically linking the extracellular domain of a suppressive receptor (in this case PD-1) to the signaling domain of an activating receptor (in this case CD28).

We benchmarked the activity of anti-MSLN TRuC-T cells coexpressing CSRs to T cells bearing only the anti-MSLN TRuC (TC-210 T cells). As it has been established that the transmembrane (TM) domain can influence the functionality of a chimeric receptor [6], we compared anti-MSLN TRuC-T cells engineered to coexpress PD1-CD28 CSRs containing either PD1TM or CD28TM. The resulting lead construct, PD1TM CSR, was integrated into a novel anti-MSLN cell therapy product designated TC-510. The efficacy of TC-510 and TC-210 were compared using an in vitro stimulation assay. Durable protection against tumor rechallenge in vivo was also assessed.

## Materials and methods

### T cell engineering

MSLN-targeting ɛ-TRuC was generated as described [1]. A PD1TM CSR was generated by isothermal assembly of the ecto- and TM domains of PD-1 (Q15116 amino acids 1–191) to the intracellular domain of CD28 (P10747 amino acids 180–220). Similarly, a CD28TM CSR was generated by isothermal assembly of the ectodomain of PD-1 (Q15116 amino acids 1–170) to the TM and intracellular domains of CD28 (P10747 amino acid 153–220). MSLN-targeting ɛ-TRuC and the CSR were cloned on the same lentivirus expression vector upstream and downstream of a T2A sequence, respectively.

Lentiviruses were prepared by transient transfection of HEK293 suspension cells with packaging plasmids and the TRuC or CAR lentiviral transfer plasmids. Supernatants were collected 48 h post-transfection, centrifuged, filtered, and precipitated. Clarified supernatants were resuspended in TexMACS medium (Miltenyi Biotech, Berisch Gladbacj, Germany) supplemented with 3% human antibody serum (Gemini Bio-Products, West Sacramento, CA) and stored at –80°C until use.

On Day 0, primary human T cells were isolated by magnetic bead separation using anti-CD4 and anti-CD8 microbeads. T cells were activated using Human T Cell TransAct (Miltenyi Biotech) at a 1:1 ratio and cultured in TexMACS medium with 3% human antibody serum (Gemini Bio-Products), 12.5 ng/mL human IL-7, and 12.5 ng/mL human IL-15 (Miltenyi Biotech). T cells were transduced with the respective lentiviral vectors on Day 1, harvested on Day 10, and frozen prior to use in functional assays.

### Cell lines

Tumor cell lines were purchased from ATCC (mesothelioma [MSTO]-211H [CRL-2081™]; Mannasas, VA) or Millipore Sigma (A2780 [C30]; St Louis, MO). For the generation of target cell lines, full-length firefly luciferase (Luc) or the PD-L1 ecto- and TM domains were cloned into pCDH-CMV-MCS-EF1a-Neo. Full-length human MSLN was cloned into pCDH- pCDH-EF1a-MCS-T2A-Puro (SBI, Palo Alto, CA), using XbaI and EcoRI restriction sites. Stably transduced cells were selected with neomycin (Millipore Sigma) and/or puromycin (Corning, Bedford, MA).

### Flow cytometry analysis

The transduction efficiency, in vitro expansion, activation/exhaustion, and proliferation of engineered T cells were analyzed by flow cytometric analysis. Cells were stained using fluorescently labeled antibody cocktails, and data were acquired on the BD LSR Fortessa™ X-20 cell analyzer. Data analysis was performed using FlowJo software (TreeStar Inc, Ashland, OR). Detailed methods are provided in the supplemental material.

### Luciferase activity-based tumor cell cytotoxicity assay

Luciferase-expressing tumor cells were plated in triplicate in a 96-well plate at 1.0 × 10^4^ cells per well, and T cells were added at the desired effector-to-target (E-to-T) ratios. After 24-h coculture, 50% of the culture supernatant was removed for cytokine analysis. Cell viability was determined using the Bright-Glo™ Luciferase Assay System (Promega, Madison, WI) according to the manufacturer’s protocol. Relative luminescence units (RLU) were measured using the SpectraMax M5 plate reader (Molecular Devices, Sunnyvale, CA). The percentage of tumor cell killing was calculated by the following formula: % tumor cell lysis = 100% × [(1 − RLU (tumor cells + T cells)/RLU (tumor cells)].

### Coculture assays

For TRuC-T cell coculture assays with target cell lines, TRuC-T cells were first thawed and rested in IL-2 (300 U/mL) for 72 h. At the end of the rest period, TRuC-T cells were then normalized for transduction efficiency and then plated in a 96-well U-bottom plate at a 1:1 ratio with 1.0 × 10^5^ Streck-treated tumor cells (Streck, La Vista, NE) for up to 96 h. Culture supernatants were harvested from replicate plates at 24 or 72 h and stored at –80 °C until sample analysis. Detailed methods, including rechallenge assay conditions, are provided in the supplemental material.

### Plate-bound MSLN and PD-L1 assay

TRuC-T cells were recovered from cryopreservation by incubation in IL-2 (300 IU/mL) for 72 h. MSLN- and PD-L1-coated 96-well ELISA microplates were prepared by treatment for 24 h with PD-L1-Fc alone (2 mg/mL) or MSLN (1 mg/mL) with varying concentrations of PD-L1-Fc (0–10 mg/mL), washed, and stored semi-dry prior to use. Recovered TRuC-T cells were normalized for transduction efficiency, and incubated at 1 × 10^5^ TRuC-T cells/well in coated-plates for 72 h. The resulting levels of IL-2, IFN-γ, TNF-α, and GM-CSF were measured using a Meso-Scale Discovery gold kit (Mesoscale Diagnostics, Rockville, MD) per the manufacturer’s instructions.

### In vivo efficacy of engineered T cells

For the subcutaneous xenograft model, 1.0 × 10^6^ MSTO-MSLN-PD-L1-Luc cells were resuspended in sterile PBS, mixed 1:1 with ice cold Matrigel^®^ (Corning, Tewksbury, MA), and then injected subcutaneously in the dorsal hind flank of 7–8-week-old female class I/class II negative NOD scid gamma (NSG) mice (NOD.Cg-Prkdc^scid^ H-2K1^tm1Bpe^H2-Ab1^em1Mvw^H2D1^tm1Bpe^Il2rg^tm1Wj^l/SzJ) from the Jackson Laboratory (Bar Harbor, ME). Mice were randomized into treatment groups by tumor burden prior to injection of human T cells; *n* = 10 mice per group. Engineered human T cells were administered at a dose of 2.0 × 10^6^ TRuC^+^ T cells per mouse, via tail vein injection when the tumor size was 150–200 mm^3^ (Day 0). Tumor growth was monitored as tumor volume by caliper measurement twice weekly. The volume of tumor was calculated as: tumor volume = (length × width^2^)/2. For tumor rechallenge in mice that had become tumor-free, 1.0 × 10^6^ MSTO-MSLN-PD-L1-Luc cells were prepared as described above and injected subcutaneously in the opposing flank on Day 44. Data represent two independent experiments with two T cell donors.

## Results

### Phenotype of TRuC-T cells coexpressing a PD1-CD28 CSR

**Fig. 1:**
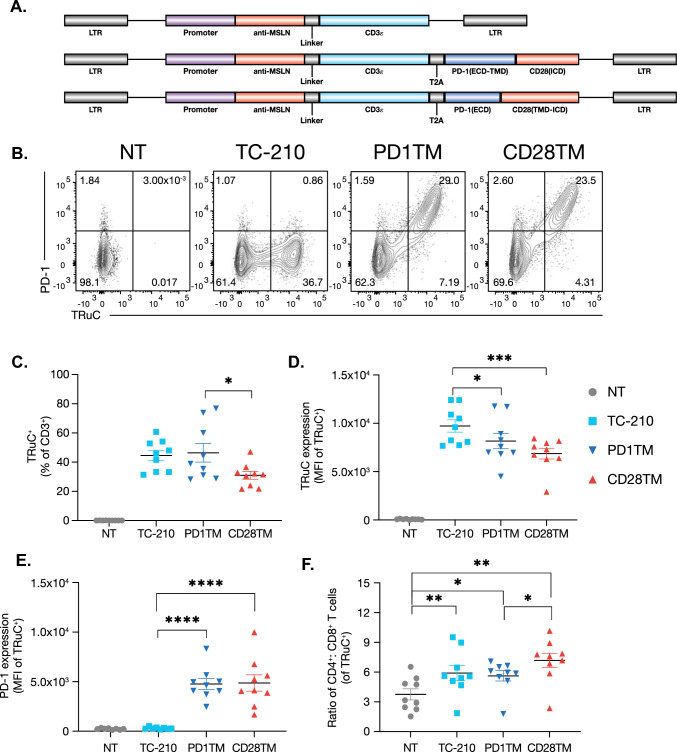
Characterization of MSLN TRuC-T cells expressing a chimeric PD1-CD28 receptor. **a** Lentiviral constructs containing the MH1 anti-mesothelin TRuC (TC-210), or with a bi-cistronic construct containing the anti-mesothelin TRuC followed by a sequence encoding the PD1-CD28 CSR with the transmembrane region of either PD-1 (PD1TM) or CD28 (CD28TM). **b** Coexpression of PD-1 and the TRuC receptor in T cells at Day 10 of expansion. **c** Percent transduction of CD3^+^ T cells as measured by TRuC receptor expression. **d** MFI of TRuC receptor expression of TRuC^+^ T cells. **e** MFI of PD-1 expression of TRuC^+^ T cells. **f** Frequency of CD8^+^ and CD4^+^ T cells TRuC^+^ T cells on Day 10 of process. Data were analyzed for statistical significance by two-way ANOVA*.* *p < 0.05, **p < 0.01, ***p < 0.001, ****p < 0.0001. Data shown are from a single experiment representing nine donors. *CD* cluster of differentiation, *CSR* chimeric switch receptor, *ECD* extracellular domain, *ICD* intracellular domain, *LTR* long terminal repeat, *MFI* median florescence intensity, *MSLN* mesothelin, *NT* nontransduced, *PD-1* programmed cell death protein 1, *TRuC* T cell receptor fusion construct, *TMD* transmembrane domain

Purified T cells were activated and transduced with a lentiviral construct expressing the anti-MSLN TRuC (TC-210) alone, or in combination with CSRs harboring the CD28TM or the PD1TM (Fig. 1a). After a 9-day expansion period, a similar transduction efficiency, as assessed by the total percentage of TRuC-expressing T cells, was observed between the TC-210 and TC-210 + PD1TM CSR. T cells with the CD28TM CSR showed a significantly lower transduction efficiency of the TRuC (Fig. 1b, c) and a reduction in median fluorescence intensity (MFI) for TRuC expression compared with TC-210 alone (Fig. 1d). T cells expressing PD1TM and CD28TM CSRs showed a similar level of PD-1 expression (mean MFI of 4870 for PD1TM and 4770 for CD28TM), indicating comparable levels of chimeric receptor expression, which were ~18-fold higher than the endogenous PD-1 levels in TC-210 alone (mean MFI of 268 in TC-210; Fig. 1e). The ratio of CD4^+^ to CD8^+^ T cells was significantly increased in all transduced groups in comparison with nontransduced (NT) controls, and the ratio was generally comparable across the transduced groups (Fig. 1f). All TRuC-T cell products showed predominantly comparable profiles with respect to memory phenotype (Fig. S1a), and the expression of activation and inhibition markers (Fig. S1b and S1c).

### In vitro functional characterization of TC-210 T cells bearing PD1-CD28 CSRs

**Fig. 2:**
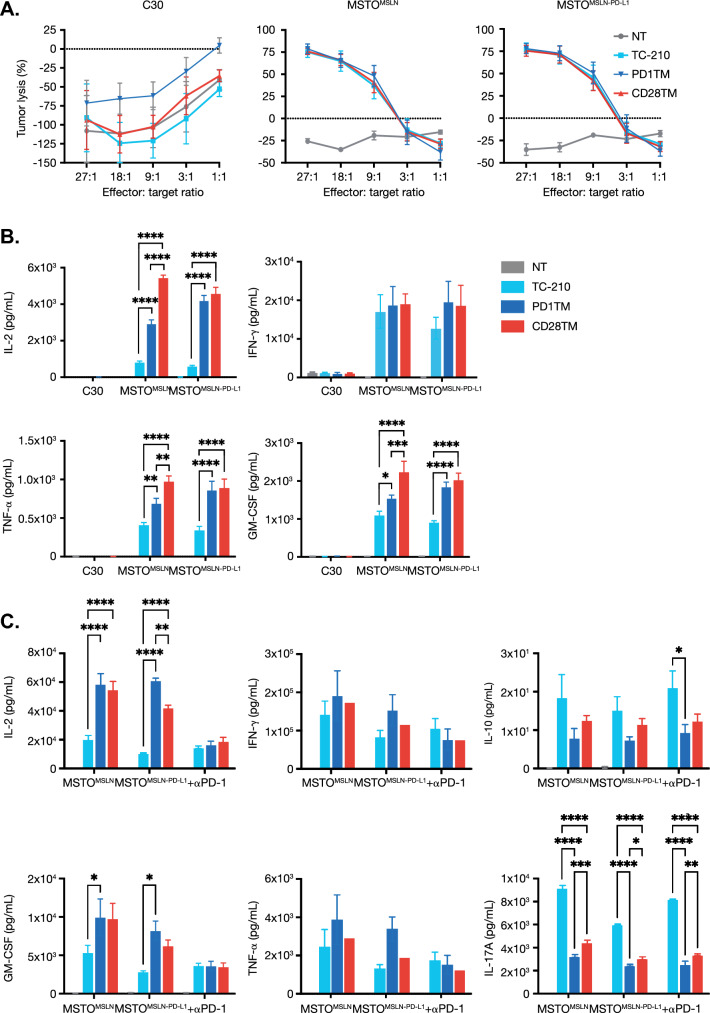
PD1-CD28 CSR enhances the function of MSLN TRuC-T cells. **a** Luc-expressing tumor cell cytotoxicity based on luminescence after 24-h coculture with MSLN TRuC-T cells at various ratios. **b** IFN-γ, IL-2, GM-CSF, and TNF-α were measured from the culture supernatants of the cytotoxicity assay by MSD ELISA. **c** Cytokine release after 24 h 1:1 coculture of MSLN TRuC-T cells with MSTO^MSLN^, MSTO^MSLN-PD-L1^, or MSTO^MSLN-PD-L1^ cells in the presence of an anti-PD-1 monoclonal antibody (+ PD-1); IFN-γ, IL-2, GM-CSF, TNF-α, IL-17, and IL-10 levels measured by MSD ELISA. Plotted data represent two or three individual donors and are plotted as mean (± SEM). Data were analyzed for statistical significance by two-way ANOVA. *p < 0.05, **p < 0.01, ***p < 0.001, ****p < 0.0001. *CSR* chimeric switch receptor, *ELISA* enzyme-linked immunosorbent assay, *GM-CSF* granulocyte macrophage colony-stimulating factor, *IFN-γ* interferon gamma, *IL* interleukin, *MSD* meso scale discovery, *MSLN* mesothelin, *MSTO* mesothelioma, *PD-L1* programmed cell death protein ligand 1, *SEM* standard error of the mean, *TNF-α* tumor necrosis alpha, *TRuC* T cell receptor fusion construct

The in vitro antitumor response of TC-210 and TC-210 + PD1-CD28 CSRs was assessed using the mesothelioma cell line, MSTO-211H, engineered to express human MSLN (MSTO^MSLN^) or MSLN and PD-L1 (MSTO^MSLN-PD-L1^). Resting TRuC-T cells were cocultured with the MSLN-negative cell line A2780 (C30), MSTO^MSLN^, or MSTO^MSLN-PD-L1^, and cytotoxicity was assessed after 24 h. Potent, antigen-specific cytotoxicity against both MSTO^MSLN^ and MSTO^MSLN-PD-L1^ was observed for all three TRuC-T cell products, with no observable differences in tumor lysis, irrespective of PD-L1 expression by the target cells (Fig. 2a).

While equivalent in their cytotoxicity, levels of cytokine secretion differed between TC-210 alone and TC-210 + PD1-CD28 CSRs, with the latter cells displaying a significantly higher level of proinflammatory cytokine (IL-2, TNF-α, and GM-CSF) production with both MSTO^MSLN^ and MSTO^MSLN-PD-L1^ target cells (Fig. 2b). Notably, the CD28TM group produced similar amounts of cytokines in response to both MSTO^MSLN^ and MSTO^MSLN-PD-L1^ targets, whereas the PD1TM group produced lower IL-2, TNF-α, and GM-CSF upon stimulation with MSTO^MSLN^ than with MSTO^MSLN-PD-L1^ cells, suggesting that the PD1TM group may require a higher PD-L1 density for full activation (Fig. 2b). We confirmed moderate endogenous PD-L1 expression in MSTO^MSLN^ and high ectopic PD-L1 expression in MSTO^MSLN-PD-L1^ (Fig. S2), demonstrating that both PD1TM and CD28TM TRuC-T cells exhibit potent cytokine responses toward tumors with either physiological or supraphysiological levels of PD-L1 expression. To confirm that increased cytokine production was mediated by CSR engagement of PD-L1, we added a PD-1-blocking antibody to the cocultures. Blockade of the CSR/PD-L1 interaction reduced proinflammatory cytokine production by CSR-bearing TRuC-T cells at least to the levels observed for TC-210 alone (Fig. 2c).

In sum, while addition of the PD1-CD28 CSRs did not increase redirected killing of tumor cells by MSLN TRuC-T cells, we observed a higher cytokine secretion and increased TCR signaling in cells bearing the CSRs.

### Regulation of PD1-CD28 CSR activation by PD-L1

**Fig. 3:**
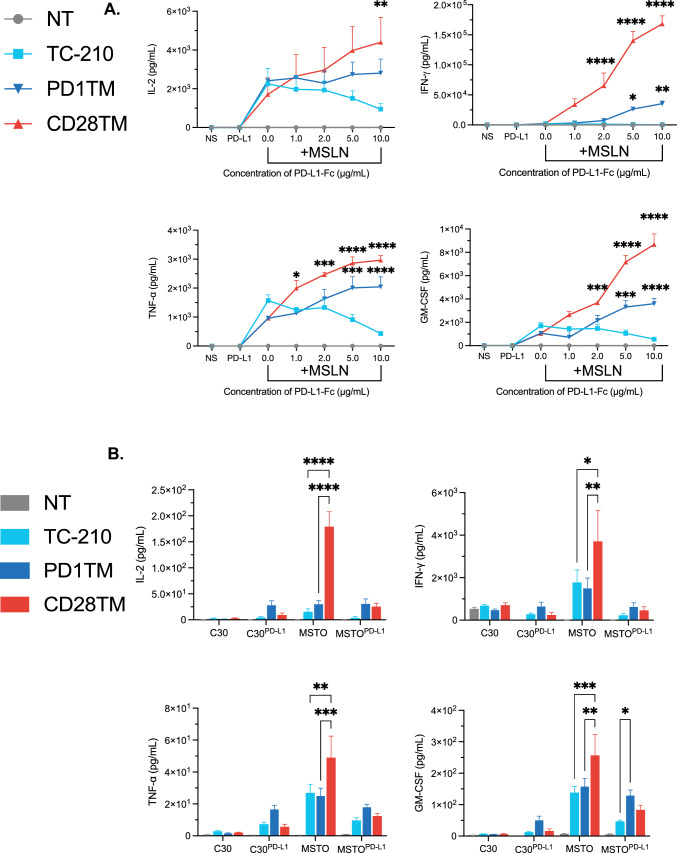
Costimulation through the chimeric PD-1 receptor is regulated by PD-L1 density. **a** MSD ELISA results from TRuC-T cells cultured with MSLN and increasing concentrations of PD-L1-Fc. **b** MSD ELISA results from MSLN TRuC-T cells cultured at a 1:1 ratio with low-MSLN antigen expressing cell lines C30, C30 overexpressing PD-L1 (C30^PD-L1^), parental MSTO, and MSTO overexpressing PD-L1 (MSTO^PD-L1^). Data were analyzed for statistical significance by two-way ANOVA. *p < 0.05, **p < 0.01, ***p < 0.001, ****p < 0.0001. Plotted data represent two or three individual donors and are plotted as mean (± SEM). *ELISA* enzyme-linked immunosorbent assay, *GM-CSF* granulocyte macrophage colony-stimulating factor, *IFN-γ* interferon gamma, *IL* interleukin, *MSD* meso scale discovery, *MSLN* mesothelin, *MSTO* mesothelioma, *NS* non-stimulated, *NT* nontransduced, *PD-L1* programmed cell death protein ligand 1, *SEM* standard error of the mean, *TNF-α* tumor necrosis alpha, *TRuC* T cell receptor fusion construct

We next assessed the sensitivity of the PD1-CD28 CSRs to increasing concentrations of plate-bound Fc-conjugated PD-L1 in the presence of a fixed concentration (1.0 μg/mL) of plate-bound MSLN that we determined to stimulate a moderate IFN-γ response (Fig. S3a). PD-L1 alone did not induce cytokine production by any TRuC-T cell (Fig. 3a), demonstrating the PD-1 CSRs adhere to the two-signal model of T cell activation. In the absence of PD-L1, MSLN antigen induced comparable levels of cytokine production by all tested TRuC-T cells. However, upon stimulation with MSLN and PD-L1, TRuC-T cells with PD1TM and CD28TM CSRs both showed increased cytokine production relative to TC-210, with the CD28TM groups consistently producing the highest levels of cytokines (Fig. 3a). For most of the cytokines measured, the PD1TM group showed a clear dose-dependent response to plate-bound PD-L1, whereas the CD28TM group was strongly activated, even at low PD-L1 levels. Furthermore, after 96 h of culture, the fold expansion of the CD28TM group peaked at 2.0 μg/mL of PD-L1 and then decreased at higher concentrations (Fig. S3b). In contrast, the PD1TM group continued to expand at higher concentrations of PD-L1 (Fig. S3b). This difference in fold expansion was associated with decreased viability of the CD28TM group (Fig. S3b and S3c).

To further compare the activation thresholds for the PD1-CD28 CSRs, we forced expression of PD-L1 in the MSLN-negative cell line C30 (C30^PD−L1^) and parental MSTO-211H cells (MSTO^PD−L1^), which express low levels of MSLN insufficient for full TRuC activation. When the MSLN TRuC-T cells were cocultured with C30 or C30^PD−L1^ cell lines, the CSR groups displayed a baseline response, further demonstrating the dependence of CSR activity on TRuC engagement. When the MSLN TRuC-T cells were cocultured with the parental MSTO cell line, the CD28TM group showed a significantly heightened cytokine response, that reduced to baseline when PD-L1 was over-expressed (MSTO^PD−L1^) (Fig. 3b). These results suggest that the CD28TM CSR sensitizes TRuC-T cells to low MSLN expression in the presence of endogenous levels of PD-L1. The heightened sensitivity of the CD28TM group for activation compared with the PD1TM group may increase the risk of cytokine release syndrome and of on-target/off-tumor toxicity. For these reasons, we selected the PD1TM CSR for integration with the anti-MSLN TRuC. We call this second-generation TRuC-T cell candidate TC-510.

### The PD1-CD28 CSR enhances TC-510 TRuC-T cell persistence in vitro in a CD28 signaling dependent manner

In addition to effector function, CD28-mediated costimulation enhances both the survival and proliferation of activated T cells. To determine if the PD1-CD28 CSR enhances the fitness of TRuC-T cells, we subjected them to an in vitro tumor rechallenge assay with MSTO^MSLN-PD-L1^ tumor cells at a low effector-to-target ratio, followed by a rechallenge every 96 h. To determine the relative contributions of PD-1 competition and CD28 costimulation to the enhanced effector function of PD1TM TRuC-T cells, we introduced previously characterized nonfunctional mutations into the CSR (PD1TM^Mutant^) [7] or deleted the CD28 signaling domain entirely (PD1^Trunc^) and verified that these constructs coexpressed well with the TRuC (Fig. S4a and S4b).

**Fig. 4:**
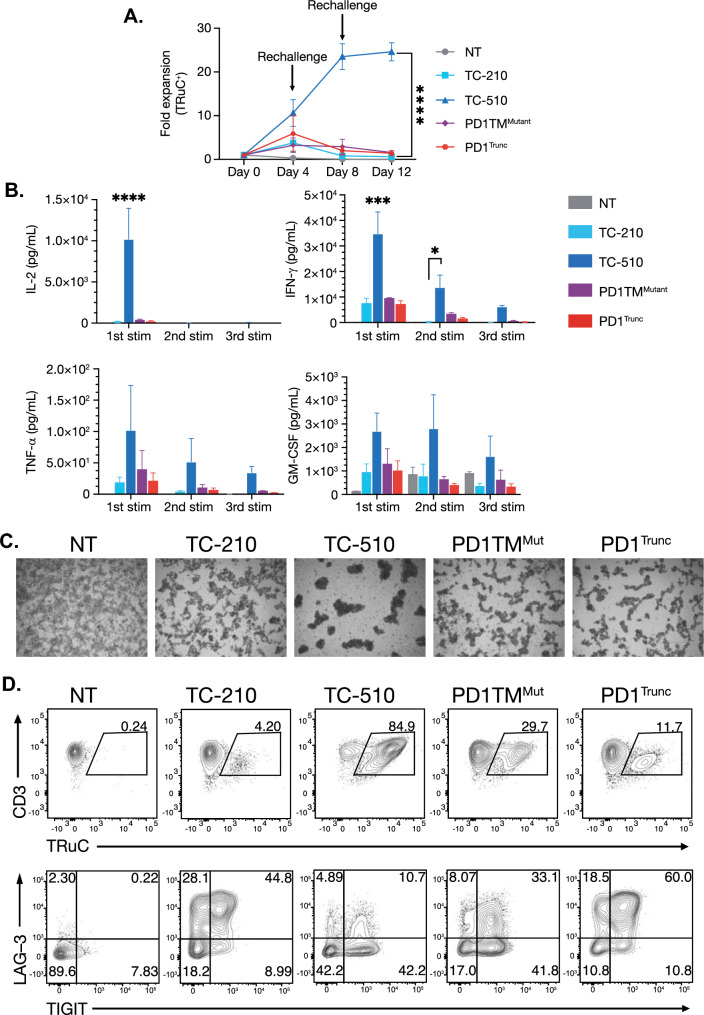
Chimeric PD-1 receptor confers enhanced fitness to TRuC-T cells during a repeated stimulation assay. **a** Fold expansion of TRuC-T cells normalized for transduction efficiency and cultured with MSTO^MSLN-PD-L1^ tumor cells at a 1:20 effector-to-target ratio. **b** MSD ELISA results from culture supernatants collected 72 h after each antigen challenge and analyzed for cytokines. **c** Brightfield microscopy images showing visible clustering in cultures containing TC-510 T cells at Day 8. **d** FACs plots of CD3 and TRuC receptor expression on viable CD45^+^ from MSTO^MSLN-PD-L1^ cultures on Day 12 of culture. Statistical analysis was carried out with a two-way ANOVA. *p < 0.05, ***p < 0.01, ****p < 0.0001. Data are representative of three independent donors and are plotted as mean (± SEM). *ELISA* enzyme-linked immunosorbent assay, *GM-CSF* granulocyte macrophage colony-stimulating factor, *IFN-γ* interferon gamma, *IL* interleukin, *LAG-3* lymphocyte activation gene 3, *MSD* meso scale discovery, *MSLN* mesothelin, *MSTO* mesothelioma, *Mut* mutant, *NT* nontransduced, *PD-1* programmed cell death protein 1, *PD-L1* programmed cell death protein ligand 1, *SEM* standard error of the mean, *TIGIT* T cell immunoglobulin and ITIM domain, *TNF-α* tumor necrosis alpha, *TRuC* T cell receptor fusion construct

TRuC-T cells normalized for transduction efficiency showed a comparable function in response to the initial antigen exposure, with no discernible differences in expansion or cytokine production between TC-210, PD1TM^Mutant^, and PD1^Trunc^ cultures (Fig. 4a, b). Following the second and third rounds of stimulation, these cultures showed contraction relative to the peak at Day 4. Examination of the culture morphology prior to the third round of stimulation revealed a diffuse pattern of cells in the TC-210, PD1TM^Mutant^, and PD1^Trunc^ culture conditions in comparison with more defined clusters of cells in the PD1TM (TC-510) cultures (Fig. 4c). Characterization of these cultures by flow cytometry revealed that the decline in expansion and cytokine production found in TC-210, PD1TM^Mutant^, and PD1^Trunc^ cultures was associated with coexpression of the exhaustion markers LAG3 and TIGIT by TRuC^+^ T cells (Fig. 4d). In contrast, the TC-510 group displayed continuous expansion over the course of the assay (Fig. 4a), with an increased and better sustained cytokine response compared with TC-210, PD1TM^Mutant^, and PD1^Trunc^ (Fig. 4b). Furthermore, TC-510 showed a less exhausted phenotype at the end of the assay (Fig. 4d).

### The PD1-CD28 CSR endows TC-510 T cells an ability to protect from tumor rechallenge in vivo

**Fig. 5:**
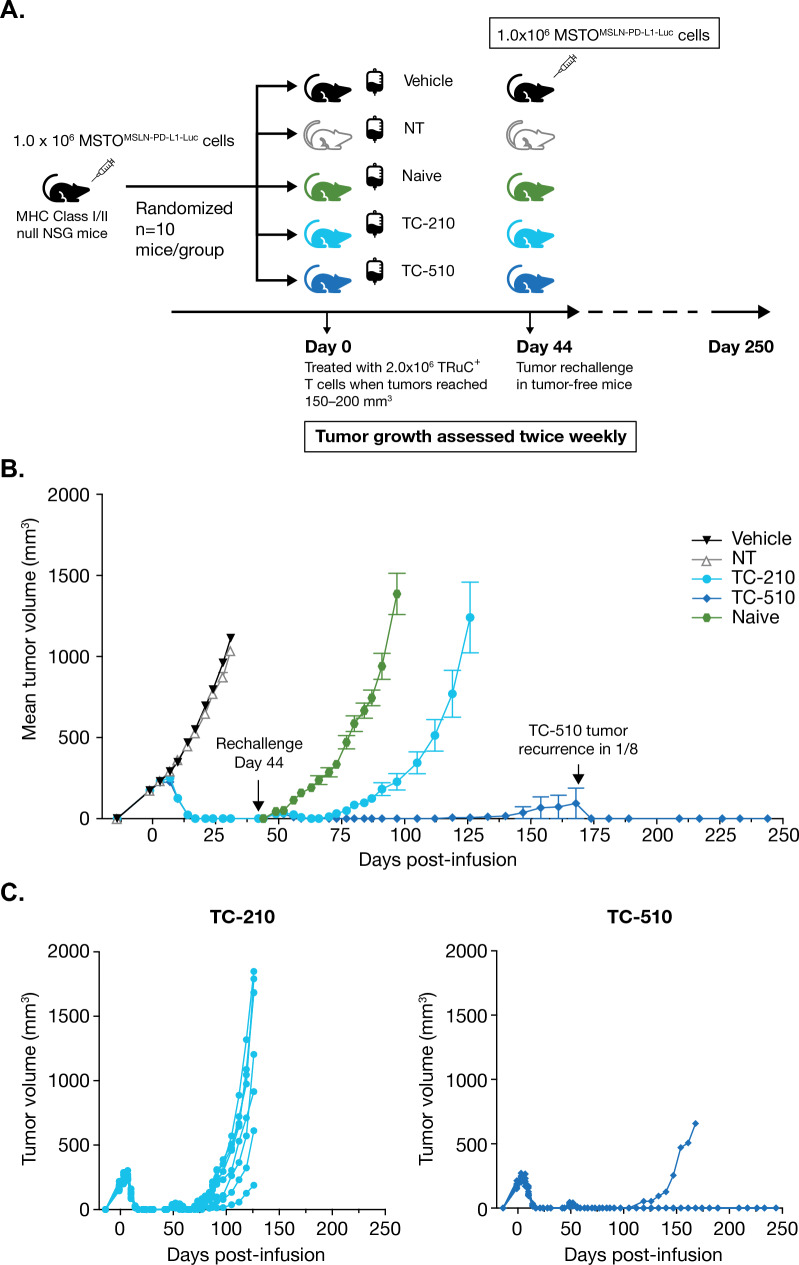
The chimeric receptor enhances in vivo efficacy of TRuC-T cells. **a** Workflow of the in vivo study design. **b** Mean tumor volume over time from NSG mice subcutaneously implanted with 1.0 × 10^6^ MSTO^MSLN-PD-L1^ tumor cells, and then treated with 2.0 × 10^6^ TRuC^+^ T cells when tumors were 150–200 mm^3^ (Day 0; *n* = 10 mice per group). On Day 44, tumor-free mice were rechallenged with 1.0 × 10^6^ MSTO^MSLN-PD-L1^ tumor cells on the opposing flank. Data are representative of two independent experiments with two donors. **c** Individual MSTO^MSLN-PD-L1^ tumor growth data from NSG mice treated with TC-210 or TC-510 on Day 0 and rechallenged with tumor cells on Day 44. Statistical analysis was carried out with a two-way ANOVA. *MSTO* mesothelioma, *NSG* NOD scid gamma, *NT* nontransduced, *PD-L1* programmed cell death protein ligand 1, *TRuC* T cell receptor fusion construct

To confirm the enhanced functionality of TC-510 TRuC-T cells in an in vivo setting, MHC Class I/II null NSG mice were subcutaneously implanted with MSTO^MSLN-PD-L1^ cells. After 14 days, when tumors had reached a volume of 150–200 mm^3^, the mice received an intravenous dose of NT, TC-210, or TC-510 TRuC-T cells (Fig. 5a). Mice treated with TC-210 and TC-510 showed comparable antitumor activity, with tumor shrinkage first evident on Day 10 post infusion and complete tumor clearance seen by Day 17 (Fig. 5b). All mice treated with TC-210 or TC-510 remained tumor-free. A tumor rechallenge was performed 44 days after T cell administration, without TC-210 or TC-510 retreatment. After a transient period of initial tumor regrowth, the mice previously treated with TC-210 or TC-510 were able to clear the rechallenge tumors; however, all the TC-210 treated mice eventually experienced tumor recurrence, whereas recurrence was limited to 1/8 mice in the TC-510 group (Fig. 5b, c). TC-510 mice that rejected the rechallenge tumors showed durable protection for the remainder of the observation period (244 days post T cell administration). This durable protection from tumor rechallenge suggests that the PD1-CD28 CSR endows TC-510 TRuC-T cells with long-term functional persistence in vivo.

## Discussion

As previously shown, TRuC-T cells differ from CAR-T cells by enabling faster tumor regression, lower cytokine production, increased tumor infiltration, and a shift toward oxidative metabolism [1]. However, like native T cells, TRuC-T cells remain susceptible to inhibition by the PD-1/PD-L1 axis [8, 9]. Furthermore, whereas engineered costimulatory signals are not required for the in vivo efficacy of TRuC-T cells, in contrast to CAR-T cells [10], we hypothesized that the delivery of costimulation in conjunction with TRuC activation would enhance T cell function and persistence. In the present study, we describe a PD1-CD28 CSR that co-opts tumor PD-L1 expression and, at the same time, drives CD28 costimulation. The potential of PD1-CD28 CSRs to improve the function of engineered T cells has previously been established in preclinical models [11–15] and in an early clinical trial [16].

Both blocking PD-1 and enhancing CD28 signaling are attractive strategies for driving tumor-targeted T cell activity and persistence in the TME. Indeed, the approved anti-PD-1 drugs have significantly changed the standard-of-care treatment in multiple cancers, improving overall survival, progression-free survival and durability of response [17, 18]. PD-1 inhibition of T cell signaling is mediated by recruitment of SHP phosphatases that deactivate TCR signaling by targeting key kinases of the TCR and CD28 signaling pathways [19, 20], and CD28 itself is a target of PD-1-activated SHP phosphatases [21]. Regardless of PD-1-mediated inhibition, activation of CD28 by ligation to B7.1 and B7.2 on antigen-presenting cells provides a costimulatory signal that can regulate and augment endogenous TCR signaling [22–24]. CD28 phosphorylation potentiates T cell activation, leading to enhanced proliferation, effector function, and notably induction of proinflammatory cytokines, such as IL-2 [25–27].

The selection of the TM domain utilized in a chimeric receptor can have a profound effect on its function. To our knowledge, we were first to functionally compare PD1-CD28 CSRs utilizing either a PD1TM or CD28TM. Although both versions of the CSR provided a CD28 signaling enhancement to TRuC-T cells, there were potentially important functional differences between them. The CD28TM failed to discriminate between low and high PD-L1 expression density on target cells and demonstrated an exaggerated response to low levels of PD-L1 in a plate-bound assay. Furthermore, the CD28TM consistently produced more cytokines against MSTO cells that express low levels of both MLSN and PD-L1, suggesting that the CD28TM increases TRuC antigen sensitivity when PD-L1 levels are low, but not when PD-L1 levels are high. A signaling imbalance between the TRuC and CSR in the low antigen/high PD-L1 setting may explain this observation. In contrast, the PD1TM demonstrated a greater dynamic response to PD-L1 density as it exhibited lower activation compared with CD28TM when PD-L1 density was low, but rivaled or surpassed the CD28TM when the PD-L1 density was high. Moreover, the PD1TM also maintained better T cell viability at high PD-L1 expression densities in a plate-bound assay. The stochastic sensitivity of the CD28TM to PD-L1 levels may be explained by CSR homodimerization and/or heterodimerization with endogenous CD28, resulting in amplified signaling [28]. We selected the PD1TM version of the CSR for its greater sensitivity to regulation by PD-L1 levels, which we believe reduces the risk of cytokine release syndrome and on-target/off-tumor toxicity, and integrated this with the anti-MSLN TRuC utilized in TC-210 to create a second-generation TRuC-T cell product that we call TC-510.

Like TC-210, cytotoxicity and cytokine release in TC-510 cells are dependent upon TRuC engagement by MSLN. This indicates that the costimulatory activity of the PD1-CD28 CSRs adheres to the two-step model of T cell activation [29], which is an important feature in the context of off-tumor toxicity risk.

The PD1-CD28 CSR contained in TC-510 offers two potential mechanisms for TRuC-T cell enhancement: (1) acting as a PD-1 dominant-negative receptor (DNR); and (2) delivering a costimulatory signal upon PD-L1 engagement. To elucidate the relative contributions of these two mechanisms, we compared TC-510 with T cells bearing the same TRuC but with the PD1TM CSR replaced by either a truncated PD-1 lacking an intracellular signaling domain (PD1^Trunc^) or a PD1TM CSR in which CD28 signaling was mutationally inactivated (PD1TM^Mutant^). TRuC-T cells coexpressing either of these constructs failed to enhance the activity of TC-510; rather, they performed comparably to TC-210. These findings support the assertion that CD28 costimulatory signaling is the primary mechanism by which the PD1TM CSR enhances TC-510 effector function. The lack of TRuC-T cell functional enhancement by the PD-1 DNR seems unexpected but is consistent with prior observations [30]; however, others have reported CAR-T cell enhancement by a PD-1 DNR [31]. This may reflect limitations in the ability of our assays to detect PD-1-mediated suppression or an intrinsic resistance of TRuC-T cells to PD-1-mediated suppression.

Our observation of enhanced expansion and persistence of TC-510 upon serial tumor rechallenge in vitro was further supported by an in vivo study in which TC-510 showed a superior ability to durably protect mice from tumor rechallenge compared with TC-210. This result indicates that integrating a PD1-CD28 CSR into TRuC-T cells enhances their capacity for improved persistence both in vitro and in vivo*,* which could translate into improved clinical efficacy in cancer patients with solid tumors. Understanding the potential impact of increased activation and persistence mediated by the CSR on safety will be an important focus of planned clinical studies.

Potential limitations of these preclinical studies include the artificial, nonphysiological nature of the in vitro assays used to assess T cell functionality and the use of a xenograft mouse model lacking both an intact immune system and expression of human PD-L1 or MSLN on normal tissues.

Based on these promising preclinical findings, TC-510 is currently being evaluated in a Phase 1/2 clinical trial in patients with advanced MSLN-expressing solid tumors (NCT05451849).

### Supplementary Information

Below is the link to the electronic supplementary material.

Supplementary file1 (DOCX 581 kb)

## Data Availability

The authors confirm that the data supporting the findings of this study are available within the article and/or its supplementary materials.

## References

[1] Ding J, Guyette S, Schrand B (2023). Mesothelin-targeting T cells bearing a novel T cell receptor fusion construct (TRuC) exhibit potent antitumor efficacy against solid tumors. OncoImmunology.

[2] Driessens G, Kline J, Gajewski TF (2009). Costimulatory and coinhibitory receptors in anti-tumor immunity. Immunol Rev.

[3] Han Y, Liu D, Li L (2020). PD-1/PD-L1 pathway: current researches in cancer. Am J Cancer Res.

[4] Beyersdorf N, Kerkau T, Hunig T (2015). CD28 co-stimulation in T-cell homeostasis: a recent perspective. Immunotargets Ther.

[5] Schwartz RH (2003). T cell nergy. Annu Rev Immunol.

[6] Guo C, Wang X, Zhang H, Zhi L, Lv T, Li M, Lu C, Zhu W (2019). Structure-based rational design of a novel chimeric PD1-NKG2D receptor for natural killer cells. Mol Immunol.

[7] Tian R, Wang H, Gish GD (2015). Combinatorial proteomic analysis of intercellular signaling applied to the CD28 T-cell costimulatory receptor. Proc Natl Acad Sci U S A.

[8] Riley JL (2009). PD-1 signaling in primary T cells. Immunol Rev.

[9] Lim AR, Rathmell WK, Rathmell JC (2020). The tumor microenvironment as a metabolic barrier to effector T cells and immunotherapy. Elife.

[10] Maher J, Brentjens RJ, Gunset G, Riviere I, Sadelain M (2002). Human T-lymphocyte cytotoxicity and proliferation directed by a single chimeric TCRzeta/CD28 receptor. Nat Biotechnol.

[11] Prosser ME, Brown CE, Shami AF, Forman SJ, Jensen MC (2012). Tumor PD-L1 co-stimulates primary human CD8(+) cytotoxic T cells modified to express a PD1:CD28 chimeric receptor. Mol Immunol.

[12] Ankri C, Shamalov K, Horovitz-Fried M, Mauer S, Cohen CJ (2013). Human T cells engineered to express a programmed death 1/28 costimulatory retargeting molecule display enhanced antitumor activity. J Immunol.

[13] Liu X, Ranganathan R, Jiang S (2016). A chimeric switch-receptor targeting PD1 augments the efficacy of second-generation CAR T cells in advanced solid tumors. Cancer Res.

[14] Schlenker R, Olguin-Contreras LF, Leisegang M (2017). Chimeric PD-1:28 receptor upgrades low-avidity T cells and restores effector function of tumor-infiltrating lymphocytes for adoptive cell therapy. Cancer Res.

[15] Lesch S, Nottebrock A, Rataj F, Heise C, Endres S, Kobold S (2022). PD-1-CD28 fusion protein strengthens mesothelin-specific TRuC T cells in preclinical solid tumor models. Cell Oncol (Dordr).

[16] Liu H, Lei W, Zhang C (2021). CD19-specific CAR T cells that express a PD-1/CD28 chimeric switch-receptor are effective in patients with PD-L1-positive B-cell lymphoma. Clin Cancer Res.

[17] He X, Xu C (2020). Immune checkpoint signaling and cancer immunotherapy. Cell Res.

[18] Ou SL, Luo J, Wei H, Qin XL, Du SY, Wang S, Jiang Q (2022). Safety and efficacy of programmed cell death 1 and programmed death ligand-1 inhibitors in the treatment of cancer: an overview of systematic reviews. Front Immunol.

[19] Gaud G, Lesourne R, Love PE (2018). Regulatory mechanisms in T cell receptor signalling. Nat Rev Immunol.

[20] Arasanz H, Gato-Canas M, Zuazo M, Ibanez-Vea M, Breckpot K, Kochan G, Escors D (2017). PD1 signal transduction pathways in T cells. Oncotarget.

[21] Hui E, Cheung J, Zhu J (2017). T cell costimulatory receptor CD28 is a primary target for PD-1-mediated inhibition. Science.

[22] Azuma M, Ito D, Yagita H, Okumura K, Phillips JH, Lanier LL, Somoza C (1993). B70 antigen is a second ligand for CTLA-4 and CD28. Nature.

[23] Freeman GJ, Freedman AS, Segil JM, Lee G, Whitman JF, Nadler LM (1989). B7, a new member of the Ig superfamily with unique expression on activated and neoplastic B cells. J Immunol.

[24] Gimmi CD, Freeman GJ, Gribben JG, Sugita K, Freedman AS, Morimoto C, Nadler LM (1991). B-cell surface antigen B7 provides a costimulatory signal that induces T cells to proliferate and secrete interleukin 2. Proc Natl Acad Sci USA.

[25] Esensten JH, Helou YA, Chopra G, Weiss A, Bluestone JA (2016). CD28 costimulation: from mechanism to therapy. Immunity.

[26] Fife BT, Bluestone JA (2008). Control of peripheral T-cell tolerance and autoimmunity via the CTLA-4 and PD-1 pathways. Immunol Rev.

[27] Rohrs JA, Siegler EL, Wang P, Finley SD (2020). ERK activation in CAR T cells is amplified by CD28-mediated increase in CD3zeta phosphorylation. iScience.

[28] Leddon SA, Fettis MM, Abramo K, Kelly R, Oleksyn D, Miller J (2020). The CD28 transmembrane domain contains an essential dimerization motif. Front Immunol.

[29] Ledbetter JA, Imboden JB, Schieven GL, Grosmaire LS, Rabinovitch PS, Lindsten T, Thompson CB, June CH (1990). CD28 ligation in T-cell activation: evidence for two signal transduction pathways. Blood.

[30] Kobold S, Grassmann S, Chaloupka M (2015). Impact of a new fusion receptor on PD-1-mediated immunosuppression in adoptive T cell therapy. J Natl Cancer Inst.

[31] Cherkassky L, Morello A, Villena-Vargas J, Feng Y, Dimitrov DS, Jones DR, Sadelain M, Adusumilli PS (2016). Human CAR T cells with cell-intrinsic PD-1 checkpoint blockade resist tumor-mediated inhibition. J Clin Invest.

[32] DeTora L, Toroser D, Sykes A, Vanderlinden C, Plunkett F, Lane T, Hanekamp E (2022). Good publication practice (GPP) guidelines for company-sponsored biomedical research: 2022 update. Ann Intern Med.

