# Supplementary material for: Functional enhancement of mesothelin-targeted TRuC-T cells by a PD1-CD28 chimeric switch receptor
Source: Cancer Immunol Immunother. 2023 Oct 18;72(12):4195–207. doi: 10.1007/s00262-023-03556-7 (PMC10700406; doi:10.1007/s00262-023-03556-7)

**SUPPLEMENTARY INFORMATION**

**SUPPLEMENTAL METHODS**

**Flow cytometry analysis**

The transduction efficiency of engineered T cells and the in vitro expansion, activation/exhaustion, and proliferation were analyzed by flow cytometric analysis.

For characterization of TRuC-T cells, samples were washed and stained with Live/Dead^TM^ Fixable Blue (Life Technologies Corporation, Carlsbad, CA) for 15 minutes at RT, and then treated with an anti-FcR reagent (Miltenyi Biotech, Miltenyi Biotech, Germany) before staining with a cocktail of antibodies (Biolegend, San Diego, CA) targeting the following surface antigens: fluorescein isothiocyanate anti-human CD3 (UCHTI), APC/Cy7 anti-human CD4 (RPA-T4), PE anti-human CD5 (UCHT2), PerCP-Cy5.5 anti-human CD8 (SK1), PE/Cy7 anti-human PD-1 (EH12.2H7), BV421 anti-human CCR7 (G043H7), and BV785 anti-human CD45RA (HI100). Some TRuC-T cells were stained with additional panels of antibodies to examine activation and exhaustion phenotypes using the following antibodies: PE anti-human CD70 (113-16) or PE anti-human TIGIT (A151A3G), PE/Dazzle^TM^ 594 anti-human CD25 (M-A251), or PE/Dazzle^TM^ 594 anti-human TIM3 (F38-2E2), PE/Cy7 anti-human CD69 (FN50), BV711 anti-human CD28 (CD28.2), and BV785 anti-human LAG-3 (11C3C65). A BUV395 anti-human CD45RO (UCHL1) antibody was obtained from BD Biosciences (San Diego, CA). To detect surface expression of the MH1 anti-mesothelin binder, an iFluor647-conjugated MonoRab™ Rabbit anti-camelid VHH antibody (Genscript, Piscataway, NJ) was used. Samples were incubated with the antibody cocktail for 25 minutes, washed, and then fixed in 4% formaldehyde for 20 minutes prior to washing and acquisition on the BD LSR Fortessa™ X-20 cell analyzer. Data analysis was performed with the FlowJo software (TreeStar Inc, Ashland, OR).

For proliferation studies, T cells were labeled with CellTraceViolet (Life Technologies Corporation, Carlsbad, CA) for 10 minutes at 37°C in PBS according to the manufacturer’s instructions. The labeling reaction was then quenched with RPMI-1640 media containing 10% fetal bovine serum prior to plating in the conditions described in the Results section.

**Coculture assays**

For TRuC-T cell coculture assays with target cell lines, TRuC-T cells were first thawed and rested in IL-2 (300 U/mL) for 72 hours. At the end of the rest period, TRuC-T cells were then normalized for transduction efficiency and subsequently plated in a 96-well U-bottom plate at a 1:1 ratio with 1.0×10^5^ tumor cells that were Streck-treated to arrest growth and stabilize antigen expression (Streck, La Vista, NE) for up to 96 hours. Culture supernatants were harvested from replicate plates at 24 or 72 hours and stored at –80°C until sample analysis.

For the antigen-rechallenge assays, TRuC-T cells were thawed and rested for 72 hours as described above, and then 5.0×10^3^ TRuC^+^ T cells were cocultured with 1.0×10^5^ Streck-treated tumor cells at a 1:20 effector-to-target ratio for 96 hours. At the end of every 96 hours, cultures were harvested, counted, and stained for analysis by flow cytometry. Replicate conditions were rechallenged with antigen every 96 hours by replacing the media and adding an additional 1.0×10^5^ Streck-treated tumor cells. Culture supernatants were collected at 72 hours after each challenge to examine the level of cytokine produced during the course of the assay.

The level of human cytokines in coculture supernatants was analyzed using the U-Plex Meso-Scale Discovery gold kit (Mesoscale Diagnostics, Rockville, MD) according to the manufacturer’s instructions. The four analytes that were examined for each assay were IFN-γ, IL-2, GM-CSF, and TNF-α. IL-4, IL-5, IL-10, IL-13, and IL-23 were also tested in some experiments, as described in the Results section.

**SUPPLEMENTAL FIGURES**

**Supplemental Fig. S1** Phenotype of MSLN TRuC-T cells at Day 10. **a** Memory-effector populations of total TRuC^+^ T cells. **b** TRuC-T cells from five of the nine donors stained for additional phenotypic markers of T cell activation and exhaustion and subjected to flow cytometric analysis. **c** Expression of surface expression markers CCR7, CD25, CD28, CD45RA, CD69, CD70, LAG-3, and TIM-3 represented as the percent of TRuC^+^ T cells and MFI of TRuC^+^ T cells. Statistical analysis was carried out using a two-way ANOVA. **p*<0.05, ***p*<0.01. Data are representative of the five donors used in Fig. 1. CCR7, CC-chemokine receptor type 7; CD, cluster of differentiation; LAG-3, lymphocyte activation gene 3; MFI, median florescence intensity; NT, nontransduced; TCM, central memory T cells; TEM, effector memory T cells; TEMRA, effector memory T cells re-expresses CD45RA; TIM-3, T cell immunoglobulin and mucin domain 3; TRuC, T cell receptor fusion construct; TSCM, stem memory T cells


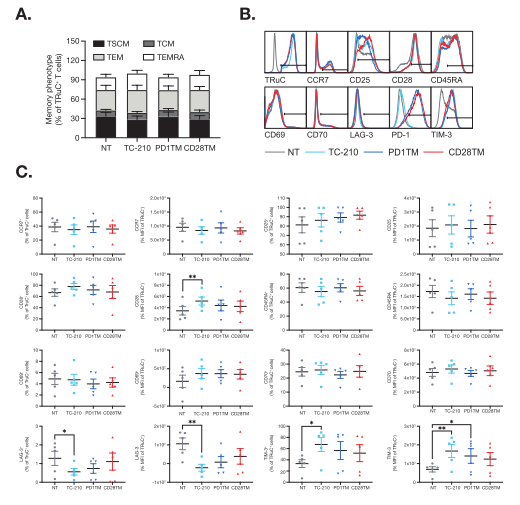


**Supplemental Fig. S2** Endogenous expression of PD-L1 in tumor cell lines during coculture with αMSLN TRuC-T cells, PD-L1 expression in parental **a** C30, Suit2, and MSTO tumor cell lines and **b** engineered cell lines MSTO-MSLN and MSTO-MSLN-PD-L1. Data are representative of a single experiment. FMO, fluorescence minus one; MSLN, mesothelin; MSTO, mesothelioma; PD‑L1, programmed cell death ligand 1; TRuC, T cell receptor fusion construct


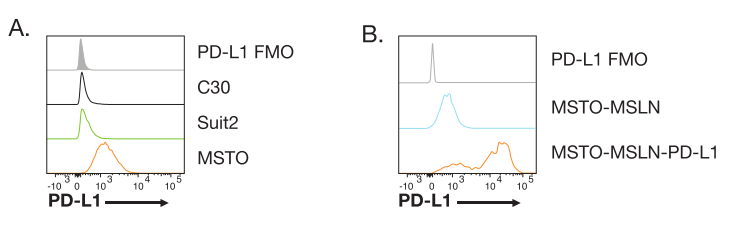


**Supplemental Fig. S3** Plate-bound MSLN cytokine release assay. **a** IFN-γ produced in response to increasing amounts of MSLN-Fc in TC-210 TRuC-T cells. **b** Fold expansion of TRuC-T cells cultured under 1.0 μg/mL of MSLN-Fc with increasing concentrations of PD‑L1-Fc at 96 hours. **c** Viability of TRuC-T cells expressed as a percentage of CD3^+^ cells. Statistical analysis was carried out with a two-way ANOVA. **p*<0.05 vs PD1TM; ***p*<0.01 vs PD1TM. CD, cluster of differentiation; IFN-γ, interferon gamma; MSLN, mesothelin;
NS, non-stimulated; NT, nontransduced; PD-1, programmed cell death protein 1;
PD-L1, programmed cell death ligand 1; TRuC, T cell receptor fusion construct

**Supplemental Fig. S4** Chimeric PD-1 receptor is dependent on receptor–ligand interaction and a functional costimulatory domain. **a** Lentiviral constructs for TC-210, or bi-cistronic vectors containing the anti-mesothelin TRuC followed by a sequence encoding PD1TM or the PD1TM CSR variants that mutate or delete the CD28 signaling domain. **b** Flow cytometry plots depicting PD-1 and TRuC receptor expression on TRuC-T cells following normalization for transduction efficiency. CSR, chimeric switch receptor; ECD, extracellular domain; ICD, intracellular domain; LTR, long terminal repeat; MSLN, mesothelin;
NT, nontransduced; PD-1, programmed cell death protein 1; TRuC, T cell receptor fusion construct


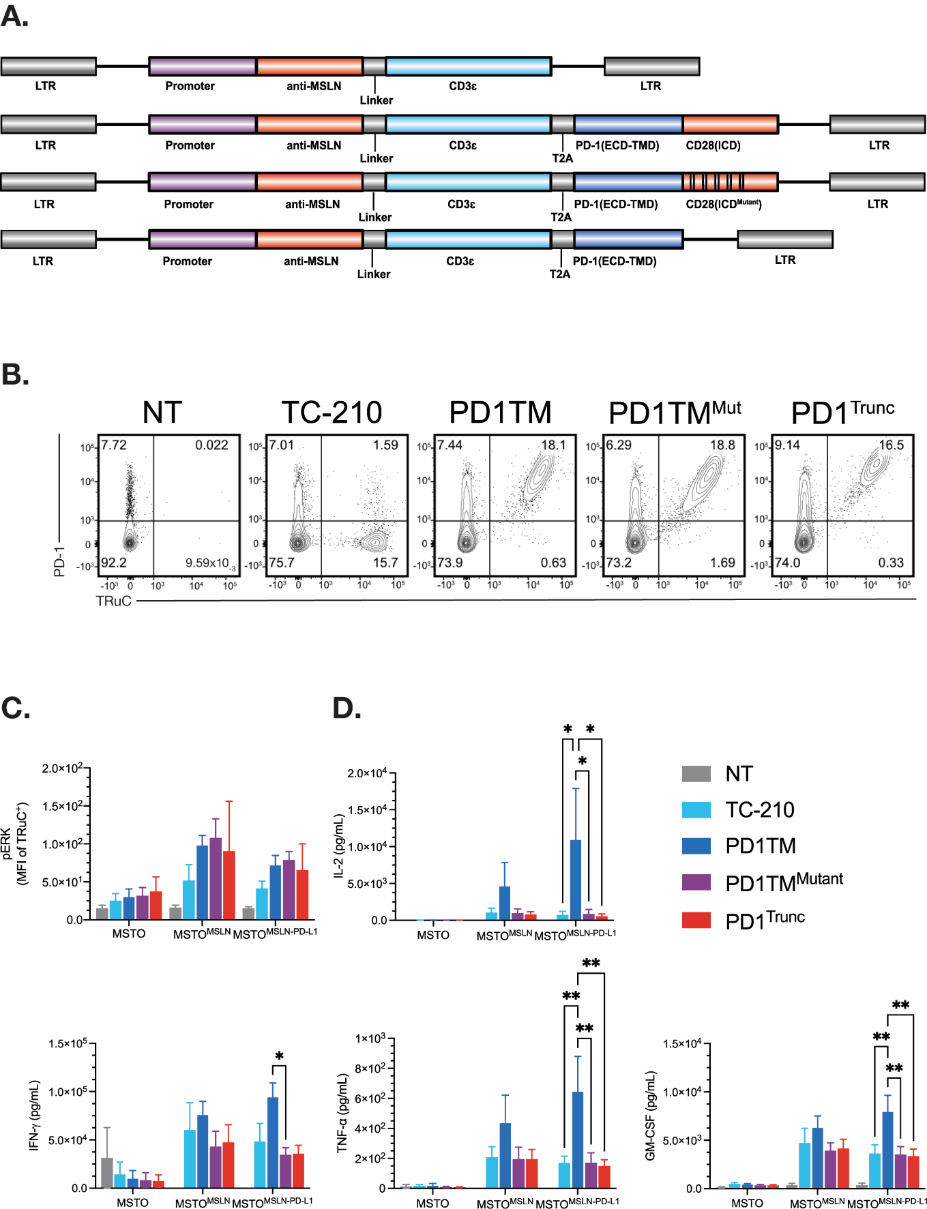

Supplement: Supplementary file 1 — Supplementary file1 (DOCX 581 kb) [file 262_2023_3556_MOESM1_ESM.docx]
